# Dr. Beddoes in Reply to Dr. Crichton

**Published:** 1799-11

**Authors:** Thomas Beddoes


### *To the Editors of the Medical and Phyſical Journal.*

GENTLEMEN,

THE etiquette of authorſhip, I ſuppoſe, requires me to anſwer the correſpondent who, in your laſt Number, maintains that he has not *falſely* repreſented me. My anſwer is reducible to two ſhort and eaſily proved propoſitions. 1. The quoted paſſages do not neceſſarily imply coincidence with Dr. GIRTANNER. 2. There has exiſted, for ſome years, incontrovertible proof that I always differed from him materially.

1. That hyperoxygenated blood is more ſtimulating, and hyperoxygenated muſcle more irritable, I truſt I have aſſiſted in rendering probable. But if the oxydable animal baſe be altered by diminution or loſs of any of its principles, the new compound, however charged with oxygene, may become leſs irritable, or altogether incapable of irritation. This was my view in penning the quoted paſſages: I had no idea of oxygen being the abſolute principle of irritability. In ſaying that "attention is not leſs due to the other elements of organized bodies," I ſurely never dreamed of apologiſing for a conjecture ſimilar to MAYOW's, relative to muſcular action.—The paſſage has nothing apologetical in ſenſe or ſound.—The conjecture needed no "kind of apology" at the time. It needs none now.

In what I firſt wrote, no man can find two oppoſite opinions. Any man might find much diſagreement with Dr. GIRTANNER; and the queſtion (if the philoſophical linguiſt will allow the diſtinction) reſpects diverſity, not oppoſition, of doctrine.

2. In your Journal (p. 203) a formal proteſt is mentioned, the term *formal* being interpolated by your correſpondent. It is true, I do not any where ſay—

"Know

"Know all men by these presents, *That I,* Thomas Beddoes, "M. D. *do disagree with* Christopher Girtanner, M. D. *in* "*manner and form following.*" But almost two years before the appearance of your correspondent's book, I wrote as follows:

"Mr. Herdman is said to have refuted the opinion that the excitability of the animal fibre depends on oxygene. Dr. Girtanner, "with whom no individual perhaps in this country has expressed his "concurrence, may indeed have been refuted. The task was truly easy. "But that the oxygene received in respiration, and distributed to the "muscles, combines with azote, hydrogene, and carbone, to form water "and various saline compounds, is a supposition which no one has yet "shewn to be at variance with fact. *The two hypotheses* differ essenti-"ally." *(Cons. on Factitious Airs, Part last, or* V. *page* 39.) The passage respecting the other elements of organized bodies is then produced; and must it not be considered as thus converted into a protest, if any thing (which I do not admit) had been before wanting to its formality? —How far your correspondent's remissness in not making himself acquainted with the latest explanations of those whom he quotes to refute, or as virtually refuted, may be culpable, or his assurance in persevering to fix upon one man the notions of another, may "equal his other "powers," it is not for me to decide.

The term false I apprehend to be often synonimous with *erroneous*—*not just.* What is hinted about *irritation* is as irrelevant as it is unfounded in fact.—Among the misfortunes which I have learned to bear without impatience, I can honestly reckon the dissent or censure of authors of works like that on *Mental Derangement.*

Your correspondent's smartness, or solemnity, I aspire not to imitate. It is not for me to summon the medical world to sit as jurors on the history or the nature of my opinions. The idle in general, and the curious about trifles, will be amused with a squabble like the present.—In my reply, and in the paper which occasioned it, they will find enough to inform their judgment; at least, I hope so; for I have done with the subject; and, in all brevity, remain,

Gentlemen,

Your's,

Oct. 4, 1799. THOMAS BEDDOES.

*Extracts*

